# Evaluation of CT Perfusion Biomarkers of Tumor Hypoxia

**DOI:** 10.1371/journal.pone.0153569

**Published:** 2016-04-14

**Authors:** Qi Qi, Timothy Pok Chi Yeung, Ting-Yim Lee, Glenn Bauman, Cathie Crukley, Laura Morrison, Lisa Hoffman, Slav Yartsev

**Affiliations:** 1 Department of Medical Biophysics, Western University, London, Ontario, Canada; 2 Robarts Research Institute, Western University, London, Ontario, Canada; 3 Lawson Imaging, Lawson Health Research Institute, London, Ontario, Canada; 4 Department of Medical Imaging, Western University, London, Ontario, Canada; 5 Department of Oncology, Western University, London, Ontario, Canada; 6 London Regional Cancer Program, London, Ontario, Canada; 7 Department of Anatomy and Cell Biology, Western University, London, Ontario, Canada; University of Modena and Reggio Emilia, ITALY

## Abstract

**Background:**

Tumor hypoxia is associated with treatment resistance to cancer therapies. Hypoxia can be investigated by immunohistopathologic methods but such procedure is invasive. A non-invasive method to interrogate tumor hypoxia is an attractive option as such method can provide information before, during, and after treatment for personalized therapies. Our study evaluated the correlations between computed tomography (CT) perfusion parameters and immunohistopathologic measurement of tumor hypoxia.

**Methods:**

Wistar rats, 18 controls and 19 treated with stereotactic radiosurgery (SRS), implanted with the C6 glioma tumor were imaged using CT perfusion on average every five days to monitor tumor growth. A final CT perfusion scan and the brain were obtained on average 14 days (8–22 days) after tumor implantation. Tumor hypoxia was detected immunohistopathologically with pimonidazole. The tumor, necrotic, and pimonidazole-positive areas on histology samples were measured. Percent necrotic area and percent hypoxic areas were calculated. Tumor volume (TV), blood flow (BF), blood volume (BV), and permeability-surface area product (PS) were obtained from the CT perfusion studies. Correlations between CT perfusion parameters and histological parameters were assessed by Spearman’s *ρ* correlation. A Bonferroni-corrected *P* value < 0.05 was considered significant.

**Results:**

BF and BV showed significant correlations with percent hypoxic area *ρ* = -0.88, P < 0.001 and *ρ* = -0.81, P < 0.001, respectively, for control animals and *ρ* = -0.7, *P* < 0.001 and *ρ* = -0.6, *P* = 0.003, respectively, for all animals, while TV and BV were correlated (*ρ* = -0.64, *P* = 0.01 and *ρ* = -0.43, *P* = 0.043, respectively) with percent necrotic area. PS was not correlated with either percent necrotic or percent hypoxic areas.

**Conclusions:**

Percent hypoxic area provided significant correlations with BF and BV, suggesting that CT perfusion parameters are potential non-invasive imaging biomarkers of tumor hypoxia.

## Introduction

Glioblastoma multiforme is the most aggressive and most common form of adult brain tumors [1]. Malignant gliomas are highly invasive tumors that invade through the process of angiogenesis [2]. Normal human brain blood vessels are very structured and well perfused. On the contrary, Tumor vessels are known to be tortuous and leaky vessels, which leads to a high interstitial fluid pressure (i.e. edema) [3,4]. This high interstitial fluid pressure impedes with oxygen delivery, and increases tumor hypoxia [5]. Tumor hypoxia is a promoter of tumor angiogenesis [2]. Therefore, tumor perfusion and tumor hypoxia are intricately related, and evaluating tumor perfusion could be a surrogate biomarker [6,7] of tumor hypoxia.

Information about tumor hypoxia is of significant clinical interest as hypoxia is known to increase resistance to radiation treatment [8]. The hypoxic regions in the tumor can be stained immunohistochemically using a hypoxia marker called pimonidazole [9]. It is a 2-nitro-imidazole compound that binds to thiol groups when the partial pressure of oxygen is below 10 mm Hg [10]. Over 65% of pimonidazole positive stained areas showed a high degree of co-localization with the PO_2_ measurement of hypoxia which is accepted as “gold standard” [11].

Perfusion can be measured non-invasively using computed tomography (CT) perfusion, magnetic resonance (MR) perfusion, and positron emission tomography (15H_2_O PET). The relationship between MR perfusion measurements and pathological measurements of tumor hypoxia have been reported in the literature [2,4], but the findings were not consistent [12–14]. PET is considered as the gold standard for measuring perfusion; however, it is not commonly used due to the short half-life of 15H_2_O (approximately 2 minutes). All three perfusion imaging techniques acquire multiple images after an injection of contrast to monitor the wash-in and wash-out of the contrast. These processes can be modeled with tracer kinetic analysis. Most importantly, quantitative mapping of blood flow (BF), blood volume (BV) and permeability-surface area product (PS) can be obtained in one CT perfusion imaging session [15]. The purpose of this study is to evaluate the correlations between CT perfusion measurements (BF, BV, and PS) and extent of tumor hypoxia on histology.

## Materials and Methods

This project was approved by the University Council on Animal Care (Project #2010–009) at Western University.

### C6 glioma model

Male Wistar rats (Charles River, Canada, age 8 to 10 weeks at surgery) weighing 300–400 g (*N* = 37) were used in this study. The animals were anaesthetized with 2% isoflurane throughout the study. C6 glioma cells (CCL-107, American Type Culture Collection, Manassas, VA) were cultivated in F12k 15% horse serum, 2.5% bovine serum, and 1% penicillin-streptomycin.

Animals were placed into a stereotactic surgical frame during C6 glioma cells implantation. The bregma was exposed after a scalp incision; a 1 mm diameter burr hole was drilled at 1 mm anterior and 3 mm right of the bregma. A total of 10^6^ C6 glioma cells were slowly injected over a period of 5 minutes at a depth of 3–4 mm from the skull surface. The burr hole was sealed with bone wax, and the scalp was closed with sutures.

### CT perfusion imaging

The CT perfusion imaging was performed on a clinical CT scanner (Discovery 750 HD, GE Healthcare, Waukesha, WI) according to previous work [16]. A two-phase CT perfusion scan, guided by a prior non-contrast CT scan that identified sixteen 1.25 mm thick sections to cover the entire brain, was performed for each animal. The brain was scanned with a high-resolution mode for 32 s at 1.4 s intervals during the first phase and for a period of 165 s at 15 s intervals during the second phase. A bolus of contrast (Isovue, Bracco Diagnostics Inc., Vaughan, Canada, 300 mg iodine/ml, 2.5 ml/kg body weight) was injected into the lateral tail vein at a rate of 0.13 ml/s at 3–4 s after the start of the first phase. The scanning parameters were 80 kVp, 120 mAs, 10 cm field of view, and high-definition bone filter. The visibly distinguishable spatial resolution was 1 line pair per 500 mm measured on a rat-size phantom [17]. The changes in CT numbers as a function of time can be measured using the dynamic series of CT perfusion images. After glioma cells implantation, baseline images were obtained on an average of 11 days (7–16 days) when the diameter of the tumor reached 4 mm [16]. Stereotactic radiosurgery (SRS) is an effective technique that delivers one or a few high dose (8–30 Gy) radiation per fraction [18,19] Rats were randomly assigned to control (*N* = 18), acute response to SRS (Group SRS1, *N* = 6), and serial imaging after SRS (Group SRS2, *N* = 13) groups. Rats in the control group were imaged every 5 days (averaged, 1–7 days) and final CT perfusion images were acquired on average 14 days (8–22 days) after the implantation of C6 glioma cells. When the diameter of the tumor reached 4 mm, rats in SRS1 and SRS2 groups underwent stereotactic radiosurgery with 12 Gy in a single fraction using helical tomotherapy (Hi-ART, v. 4.2, Accuray Inc., Sunnyvale, CA). For SRS1 group subjects, a final CT perfusion imaging was performed 5 days after radiosurgery. The 13 animals in SRS2 group were imaged up to 59 days post-SRS. Rats in SRS2 group were euthanized when the following symptoms occurred: weight loss greater or equal to 15% from the heaviest weight recorded, loss of appetite, physical disorders such as weakness on one side of the limb. On the last day of imaging, the animals were injected with pimonidazole (HP2-100, Chemicon International, Inc., Temecula, CA) at a concentration of 60 mg/kg of body weight intravenously 90 min prior to euthanasia.

### Perfusion image analysis

**Fig 1:**
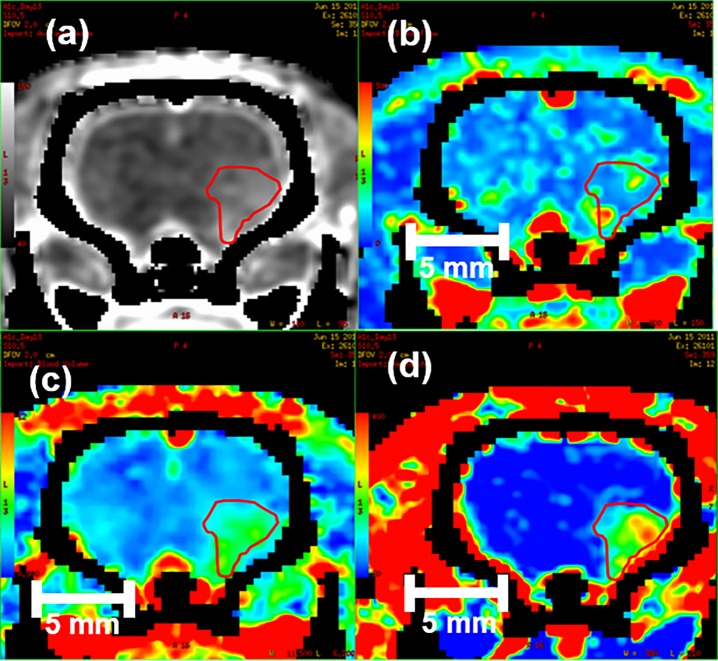
Delineation of tumor and CT perfusion data. (a) Tumor. (b) Tumor blood flow (BF). (c) Tumor blood volume (BV). (d) Tumor permeability-surface area (PS) product.

Prototype version of CT Perfusion 4D (GE Healthcare) was used to calculate maps of BF, BV, and PS. The time attenuation curve (TAC) from the carotid artery was selected as the arterial input. The arterial TAC was deconvolved with tissue TACs measured from 2×2 pixel blocks of CT images using the Johnson-Wilson model to calculate maps of BF, BV, and PS [15]. The tissue-enhancement curve can be expressed as the convolution (⊗) between the blood flow-scaled impulse residue function (IRF), (*BF·R(t)*), and the arterial TAC, (*C*_*a*_*(t)*). The shape of the BF-scaled IRF has two distinct phases, and it is solved by devolving the arterial TAC with the tissue-enhancement curve. The plateau of the BF-scaled IRF defines the BF, while the area under the first phase of the BF-IRF is the BV. The second phase of the BF-scaled IRF starts at the height of the extraction fraction, which is the fraction of contrast agent leaks into the interstitial space. The second phase of the BF-scaled decays with time, and PS can be calculated as PS = -BF·ln(1-E). The contrast-enhancing lesion was segmented manually (Fig 1), and the mean values of BF, BV, and PS of the contrast-enhancing lesion were measured.

### Immunohistochemical staining

The animals were euthanized with an overdose of potassium chloride on the last imaging day. The animals were perfusion-fixed with phosphate buffered saline followed by 4% paraformaldehyde. The brains were removed and fixed in 4% paraformaldehyde for 24 hours. The brain specimens were sectioned into 3 mm thick blocks, paraffin-embedded, sectioned at 5 μm [10] in the same orientation as in the CT scan and picked up on positively charged microscope slides and dried overnight at 37°C. The brain sections were deparaffinized in xylene, hydrated through graded alcohols and washed in water. The slides were then placed in a bath of 3% H_2_O_2_ for 10 minutes to block peroxidase activity, washed in running tap water for 5 minutes, rinsed in distilled water and placed in pH 7.4 PBS (phosphate buffered saline), prepared by mixing 8 g of NaCl, 0.2 g of KCl, 1.44 g of Na2HPO4 and 0.24 g of KH2PO4 in 1000 ml distilled water. The slides were laid out in an incubation chamber, and the sections were covered with blocking serum (2.5% normal horse serum, ImmPRESS kit, Vector Laboratories, Inc., Burlingame, CA) and incubated for 30 minutes. The blocking serum was then carefully drained from the sections and primary antibody (anti-pimonidazole mouse IgG monoclonal antibody, Hypoxyprobe, Inc.) at a dilution of 1:1000 in 2% normal horse serum was applied, the sections were then incubated overnight at 4°C. The next morning the slides were washed 3 x 5 minutes with PBS and secondary antibody (anti-mouse IgG, ImmPRESS kit, Vector Laboratories, Inc., Burlingame, CA) was applied. The slides were incubated 40 minutes at room temperature. The slides were then washed 3 x 5 minutes with PBS before DAB (3,3’-diamiobenzidine, Vector Laboratories, Inc.) was applied for 8 minutes. The slides were then washed in running tap water, counterstained with Carazzi’s haematoxylin for 1 minute, washed with tap water, dehydrated through graded alcohols, cleared in xylene and mounted.

### Histological analysis

**Fig 2:**
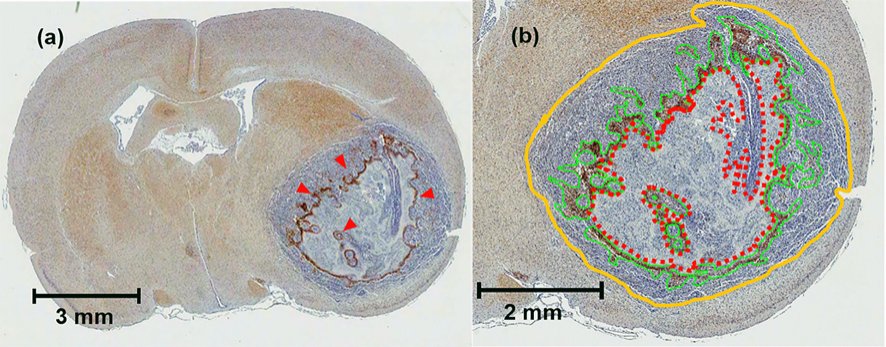
Examples of brain histology with pimonidazole. (a) 5 μm thick brain specimen stained for hypoxia (red arrows). (b) Zoomed area around the tumour (orange outline), pimonidazole-positive area (green outline), and necrotic area (red dashed line).

Brian slices were scanned using Aperior Vista ScanScope® (Leica Biosystems, CA), tumor area, hypoxic area and necrotic area of each slice were manually delineated as shown in Fig 2 using Aperio ImageScope software (v.12.1, Leica Biosystems, CA). The extent of hypoxia for each slice was measured by calculating the percent hypoxic area:

$$\%\;\text{Hypoxic Area}=\frac{\text{hypoxic area}}{\text{tumor area}}\times 100\%$$ (1)

The extent of necrosis for each slice was measured by calculating the percent necrotic area:

$$\%\;\text{Necrotic Area}=\frac{\text{necrotic area}}{\text{tumor area}}\times 100\%$$ (2)

The average percent hypoxic and average necrotic areas of an animal were calculated by averaging over all brain slices containing tumor cells.

### Statistical analysis

The normality of data was inspected using Shapiro-Wilk test. The correlation between CT perfusion parameters and histology measurement of hypoxia and necrosis were evaluated using IBM SPSS version 22 by the Spearman’s rank correlation. A *P* value ≤ 0.05 was considered as statistically significant.

## Results

**Fig 3:**
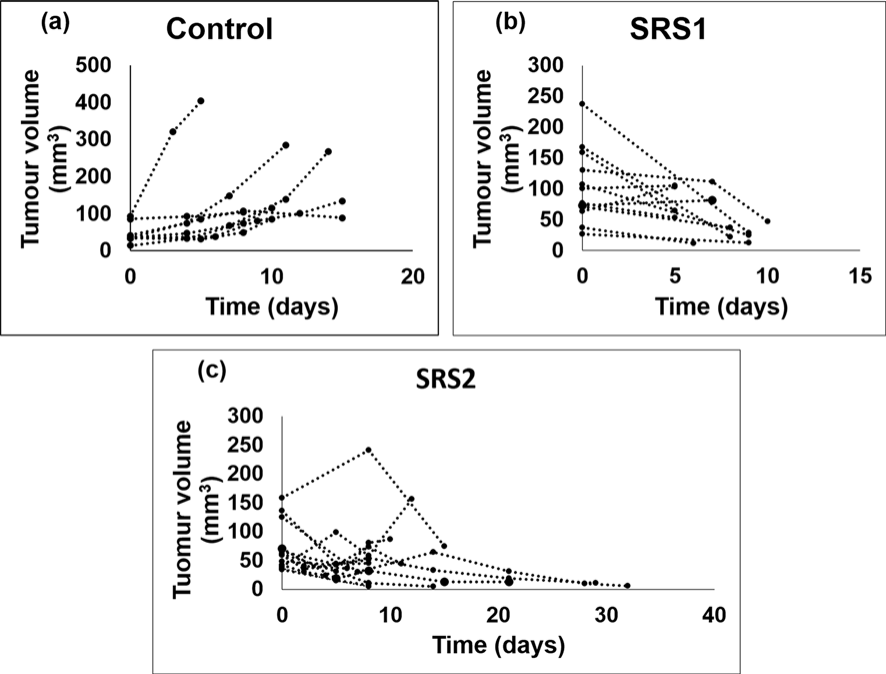
Tumor volume vs. time for animals in (a) control, (b) SRS1, and (c) SRS2 groups.

Tumor volume changes observed on CT images are shown in Fig 3 for representative examples from different groups. Time = 0 corresponds to the day when the diameter of the tumor reached 4 mm for all groups. We observed a steady increase of tumor volume for all animals in the control group, but the growth rate was quite diversified as shown for typical examples in Fig 3A. Delivery of 12 Gy in a single fraction drastically reduced tumor volumes in both SRS1 and SRS2 groups. In SRS1 group, the tumor shrinkage was faster for larger initial lesions: Fig 3B. Interestingly, in three cases the tumor shrinkage in the SRS2 group occurred with a delay (Fig 3C), and in four subjects out of 7 tumors disappeared completely.

The number of positive immunohistochemistry staining in control, SRS1, and SRS2 groups is given in Table 1. Tissue loss during buffer washing and transfer led to unsuccessful staining; a warm water bath for the sectioned sample could be essential for the success of the experiment [9].

**Table 1:** Summary of histological results.

Group	Number of rats	Positive staining	Tumor present
Control	18	13	13
SRS1	6	6	6
SRS2	13	7	3

**Fig 4:**
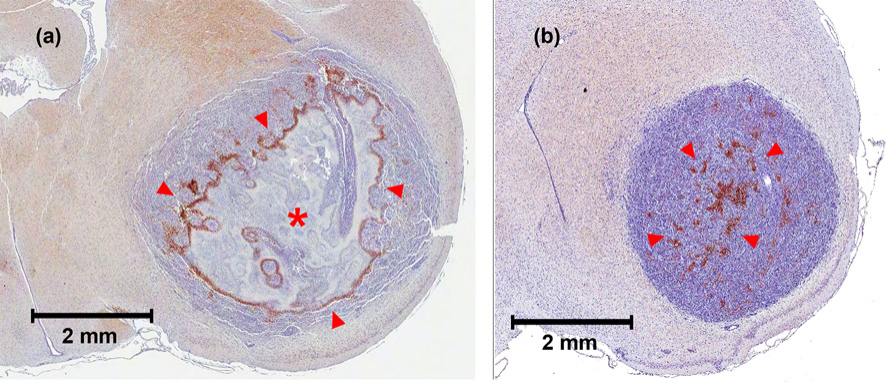
Examples of pimonidazole staining patterns. (a) Pimonidazole staining (red arrows) on the periphery of necrotic regions (red asterisk). (b) Pimonidazole staining located in the center of the tumor when there is no dominant necrotic region.

**Fig 5:**
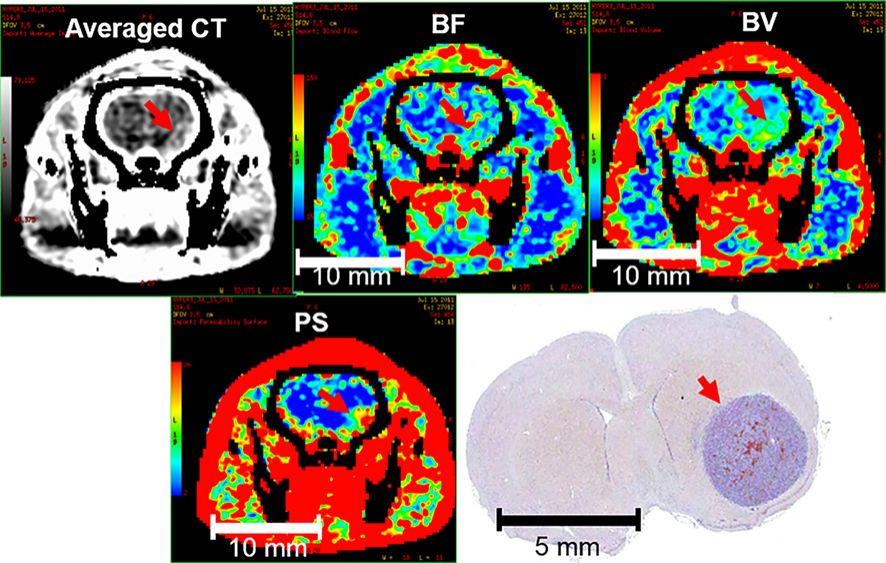
Location of the tumor (red arrows) observed in CT perfusion imaging and in histology.

When a necrosis, denoted by a red asterisk in Fig 4A, was present inside of the tumor, the hypoxic regions were commonly located at the peripheral of the necrosis as shown by the red arrows. When necrosis was absent inside of the tumor, the hypoxic regions were typically located at the center of the tumor as illustrated by the red arrows in Fig 4B. Negative staining subjects were excluded from this study. The tumors observed in CT perfusion showed a co-localization with the histology as illustrated in Fig 5.

The strength of correlation between CT perfusion parameters and extent of tumor hypoxia and necrosis for the control group (*N* = 13) is presented in Table 2. The correlations between CT perfusion parameters and extent of either tumor hypoxia available in the histology data for animals in SRS1 (*N* = 6) and SRS2 (*N* = 3) groups had the trend similar to the results in the control group. In the SRS1 group, the short-term effect of radiation on the vasculature was variable due to a large discrepancy in tumor volumes and a variety of tumor shrinkage patterns shown in Fig 3B. In the SRS2 group, where the animals were observed for a relatively long time after irradiation, the tumor disappeared completely in four out of 7 animals, so that only three animals had tumor left for histology study.

**Table 2:** Spearman’s rank correlations between CT perfusion parameters and histology measurement for control group (N = 18) animals.

	% Hypoxic area		% Necrotic area	
	ρ	*P*	ρ	*P*
BF (ml/min/100g)	-0.88	< 0.001	-0.78	< 0.001
Tumor volume (mm^3^)	0.65	0.017	0.81	< 0.001
BV (ml/100g)	-0.81	< 0.001	-0.64	0.019
PS (ml/min/100g)	-0.12	0.694	-0.12	0.699

**Fig 6:**
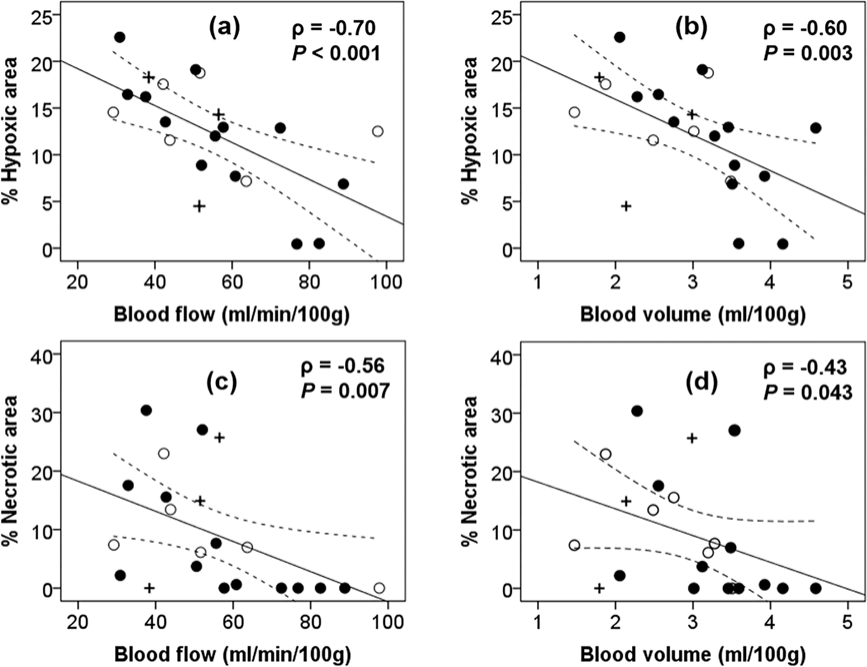
Correlation between CT perfusion parameters with hypoxia and necrosis. Percent hypoxic area vs. tumor blood flow (a), blood volume (b), percent necrotic area vs. tumor blood flow (c) and percent necrotic area vs. blood volume (d). Data for animals in control (●), SRS1(○) and SRS2(+) groups are all included. Solid lines show the best fit and dotted lines denote the 95% confidence intervals.

Although we observed a strong dependence of the percent hypoxic area on BF, the number of subjects was not sufficient for statistically sound conclusions (*P*-values for these groups are greater or equal to 0.072). The correlations between the CT perfusion parameters (BF and BV) and histology evaluated hypoxia and necrosis shown in Fig 6 for all animals from control and irradiated groups are weaker than those for control group only presented in Table 2.

As a predictor of hypoxia, blood volume parameter was not as strong as blood flow with ρ = -0.81 and -0.60, for the control group only and for the complete cohort of subjects, respectively. No significant correlations could be determined between permeability-surface area product and percent hypoxic area. Altogether, these results would likely provide evidence to support that hypoxia was driven by blood flow and not by the extent of vessel leakiness which was measured by permeability-surface area product.

**Fig 7:**
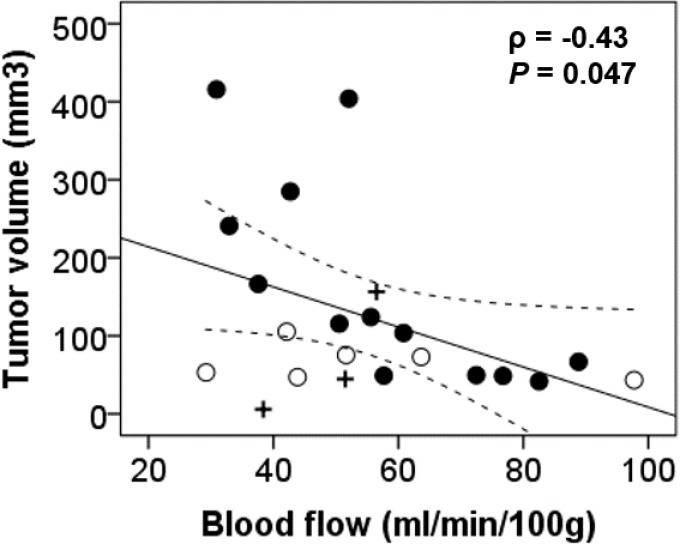
Tumor volume vs. tumor blood flow for test subjects from control (●), SRS1(○) and SRS2(+) groups.

Tumor volume and blood flow do correlate (ρ = -0.43) as shown in Fig 7, but BF is a much better predictor of hypoxia (ρ = -0.70 for both control and irradiated animals, ρ = -0.88 for the control group only) than tumor volume (ρ = 0.41 for both control and irradiated animals, ρ = 0.65 for the control group only). CT perfusion studies were also able to predict the appearance of necrosis. In the control group, blood flow strongly correlated with percent necrotic area, but the correlation for the control group was slightly weaker (ρ = -0.78) than in the case of hypoxia (ρ = -0.88). Better ability of blood flow to predict hypoxia compared to necrosis is also demonstrated by a narrower 95% confident interval in Fig 6A than in Fig 6C.

BF is not only a good representation of the extent of hypoxia and necrosis but the strength of resistance to radiation therapy [20] as well. High BF that is stimulated by angiogenesis due to high extent of hypoxia is associated with low survival after radiation treatment [20,21]. This indicates that tumor perfusion, especially BF, can provide important information for cancer treatment with radiation therapy.

## Discussion

High values of tumor BF, TV, and BV are associated with lower percent hypoxic area and lower percent necrotic area. No statistically significant correlations between PS and either percent hypoxic area or percent necrotic area were found in this study.

The overlap between perfused tissue and hypoxic tissue is an emerging topic of interest. Although a negative relationship was seen between high BF or BV and hypoxia, other studies have pointed out that tumor regions with some perfusion are the key hypoxic regions. It was demonstrated that oxygen-enhanced MRI could be used to identify perfused area that are lacking in oxygen [22]. It is important to identify perfused hypoxic area from non-perfused hypoxic area because this can help identify tumor sub-regions that are likely to resist therapies. Our study separated the perfused hypoxic area from the non-perfused necrotic area, future work should use perfusion imaging in conjunction with hypoxia imaging (oxygen-enhanced MRI or PET) to distinguish perfused hypoxic regions from non-perfused hypoxic regions.

There are a few limitations in this study that must be considered. First, the sample size for this study is small especially for histology studies in SRS1 (N = 6) and SRS2 (N = 3) groups. Second, the spatial correlation between hypoxia and CT perfusion was not considered. In order to acquire spatial correlation, measurements of partial pressure of oxygen in the brain could be performed by using a needle probe with electrochemical microsensor [11]. Third, the extent of hypoxia was approximated as an average of percent hypoxic area of different slices from the same animal. It will be more accurate if the hypoxic volume is measured which can be achieved using positron emission tomography (PET) or single-photon emission computed tomography (SPECT) [23].

## Conclusion

Our study evaluated relative significance of the CT perfusion parameters BF, BV, and PS as possible predictors of tumor hypoxia and necrosis. Tumor BF is a potential surrogate imaging biomarker of tumor hypoxia as it showed the most significant correlation with percent hypoxic area. BF and BV could also predict necrosis.
